# Paciente em Parada Cardiorrespiratória – É Possível a Realização de Implante Percutâneo de Valva Aórtica (TAVI) nesse Cenário?

**DOI:** 10.36660/abc.20201097

**Published:** 2021-08-09

**Authors:** Alexandre de Matos Soeiro, Francisco Akira Cardozo, Patrícia Oliveira Guimarães, Marcel Paula Pereira, Paulo Vinícius Ramos Souza, Gustavo A. B. Boros, Viviane Cordeiro Veiga, Samolon Soriano Ordinola Rojas, Fernanda Marinho Mangione, Salvador André Bavaresco Cristóvão, Gustavo Alexandre Dutra, Adnan Ali Salman, Luiz Eduardo Loureiro Bettarello, José Armando Mangione

**Affiliations:** 1 Unidade Cardiológica Intensiva Hospital Beneficência Portuguesa de São Paulo São PauloSP Brasil Hospital Beneficência Portuguesa de São Paulo - Unidade Cardiológica Intensiva, São Paulo, SP – Brasil

**Keywords:** Valva Aórtica, Parada Cardíaca, Emergências, Choque Cardiogênico

## Introdução

O implante percutâneo de valva aórtica (TAVI) é um procedimento bem estabelecido em centros de referência no mundo todo, sendo aceito atualmente como o método de escolha em pacientes de risco alto e intermediário. Embora incomuns, complicações catastróficas podem ocorrer antes ou após seu implante, como obstrução coronariana, rotura do anel valvar, tamponamento cardíaco, insuficiência perivalvar importante e embolização/deslocamento da prótese.

Dados de registro norte-americano publicado recentemente mostraram que 1.695 pacientes (2,8%) submetidos à TAVI necessitaram de algum tipo de suporte circulatório mecânico durante a internação. Foi observado que insuficiência cardíaca, acesso transapical, doenças respiratórias, infarto agudo do miocárdico, parada cardiorrespiratória (PCR) e choque cardiogênico foram os fatores mais associados à necessidade do suporte circulatório mecânico.^1^

Apesar de seu emprego ser difundido principalmente em pacientes de alto risco e de conhecermos as principais complicações do método e da doença de base, sua utilização na PCR ainda não é indicada e, também, até o momento, raramente descrita em literatura.^1^ O implante durante a PCR torna a técnica difícil devido às compressões torácicas, impossibilidade de avaliação do resultado imediato e tempo limitado para expansão efetiva da prótese.

Em 2013, foi descrito pela primeira vez o uso de compressão mecânica (*AutoPulse*) durante a ocorrência de PCR após início do implante da TAVI como alternativa segura e efetiva, permitindo a realização do procedimento até seu término.^2^ Já em 2014, outro relato de caso apresentou a possibilidade de deformação da prótese após reanimação cardiopulmonar (RCP) manual, evoluindo com *leak* paravalvar importante e óbito subsequente.^3^

Com base nessas possibilidades de ocorrerem complicações, existem protocolos para indicação de membrana de oxigenação extracorpórea (ECMO) em pacientes submetidos à TAVI que evoluem em choque cardiogênico ou até mesmo PCR. Em casos de alto risco, alguns serviços têm se organizado e conseguido colocar em ECMO em até 5 minutos pacientes que evoluem em PCR durante implante de TAVI. Isso permite estabilizar rápido o paciente antes da ocorrência de disfunção orgânica múltipla e até mesmo realizar o procedimento até seu ponto final.^4^

Dessa forma, os relatos de complicações e PCR associados à TAVI são descritos somente durante ou após o procedimento de expansão da endoprótese. O objetivo do presente caso é compartilhar o sucesso e o pioneirismo de um centro de referência brasileiro na realização de TAVI em paciente com PCR iniciada pré-procedimento como medida de salvamento.

## Descrição

**Figura 1:**
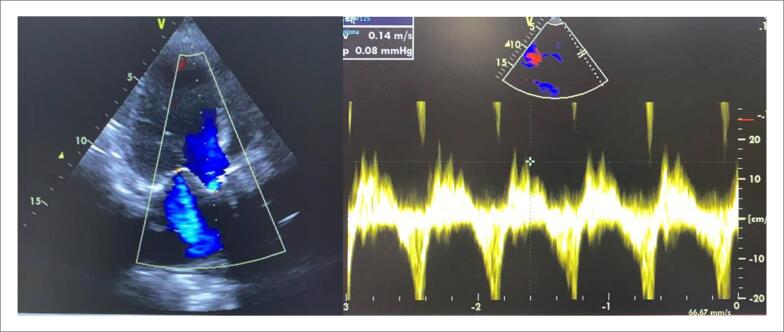
Ecocardiograma transtorácico em janela apical pré-TAVI mostrando valva aórtica calcificada com área valvar de 0,6 cm^2^ e gradiente ventrículo esquerdo-aorta máximo de 66 mmHg e médio de 35 mmHg.

Paciente de 84 anos de idade, sexo feminino, iniciou quadro de dispneia ao repouso há 6 dias. Referia antecedente pessoal de hipertensão arterial sistêmica, dislipidemia e infarto agudo do miocárdio, no qual foi realizada intervenção coronariana percutânea com *stent* farmacológico em tronco de coronária esquerda e artéria circunflexa há 2 meses. Foi inicialmente admitida via pronto-atendimento em um hospital secundário. À chegada, encontrava-se em mau estado geral, taquicárdica (frequência cardíaca = 120 bpm), taquipneica (frequência respiratória = 28 ipm), com pressão arterial de 76×40 mmHg, tempo de enchimento capilar aumentado (6 segundos), à ausculta cardíaca com bulhas rítmicas com sopro sistólico ejetivo em foco aórtico +3/+6 e murmúrios vesiculares presentes com estertores até terço médio bilateralmente à ausculta pulmonar. Foram inicialmente iniciados furosemida endovenosa, norepinefrina e dobutamina em infusão contínua, além de uso de ventilação não invasiva. Eletrocardiograma não mostrava alterações isquêmicas agudas, e marcadores de necrose miocárdica vieram negativos. *Screening* inicial também não mostrou alterações sugestivas de quadro infeccioso. Nesse momento, foi solicitado ecocardiograma transtorácico que mostrou fração de ejeção de ventrículo esquerdo de 28% com hipocinesia difusa, valva aórtica calcificada com área valvar de 0,6 cm^2^ e gradiente ventrículo esquerdo-aorta máximo de 66 mmHg e médio de 35 mmHg, além de insuficiência mitral moderada (Figura 1).

**Figura 2:**
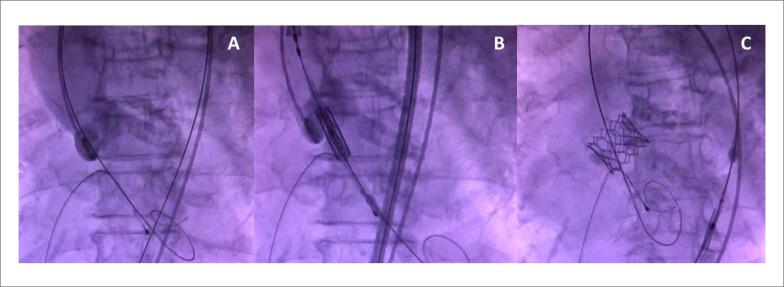
Realização de TAVI com prótese *Edward SAPIEN 3* n^o^ 26 somente com visualização da escopia durante manobras de RCP. A) Posicionamento dos cateteres. B) Alinhamento da prótese no anel valvar aórtico. C) Prótese colocada em posição aórtica.

**Figura 3:**
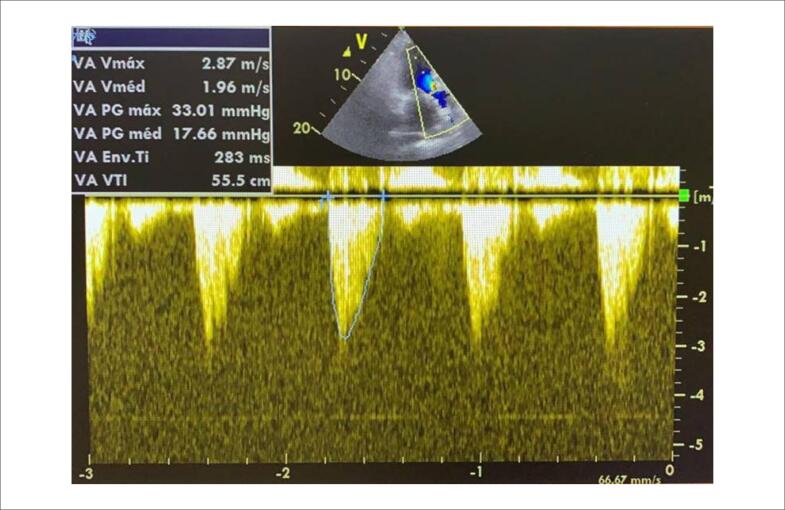
Ecocardiograma transtorácico após TAVI mostrando fração de ejeção de ventrículo esquerdo de 33%, prótese em posição aórtica com gradiente ventrículo esquerdo-aorta máximo de 35 mmHg e médio de 17 mmHg, com insuficiência aórtica periprótese de discreta a moderada.

Após duas falhas de desmame dos fármacos vasoativos foi optado por transferir a paciente em transporte aéreo intensivo para um centro terciário. Após avaliação inicial e com base no risco associado à paciente, foi indicada TAVI transfemoral. Após 72 horas no hospital de alta complexidade, a paciente evoluiu com piora do choque cardiogênico e necessidade de aumento progressivo dos fármacos vasoativos (dobutamina 20 *μ*g/kg/min e norepinefrina 0,3 *μ*g/kg/min). Dessa forma, a paciente foi transferida em caráter de urgência ao laboratório de hemodinâmica para realização do procedimento percutâneo. A paciente foi posicionada em decúbito horizontal e foi realizada a intubação orotraqueal, assepsia e obtenção do acesso femoral. Durante o início da introdução do eletrodo do marca-passo transvenoso, a paciente apresentou PCR em ritmo de fibrilação ventricular com degeneração rápida para assistolia. Foram iniciadas medidas de RCP de acordo com as diretrizes de suporte avançado de vida em cardiologia (ACLS). Nesse momento, como medida de salvamento, durante a RCP e sem auxílio do ecocardiograma, foi realizado a TAVI com prótese *Edward SAPIEN 3* n^o^ 26 somente com visualização da escopia em sala e com tempo total de 4 minutos de procedimento (Figura 2). Imediatamente após, a paciente retornou ao ritmo de circulação espontânea. No momento inicial pós-procedimento, evoluiu com piora do choque cardiogênico, com necessidade de aumento das doses de norepinefrina, uso de vasopressina e balão intra-aórtico. Novo ecocardiograma transtorácico mostrava fração de ejeção de ventrículo esquerdo de 33% com hipocinesia difusa, prótese em posição aórtica com gradiente ventrículo esquerdo-aorta máximo de 35 mmHg e médio de 17 mmHg, com insuficiência aórtica periprótese de discreta a moderada (Figura 3).

Após 48 horas, a paciente apresentou melhora dos parâmetros hemodinâmicos, sendo realizado o desmame progressivo do suporte mecânico e de fármacos vasoativos e submetida à extubação no sétimo dia após a TAVI, permanecendo sem déficits neurológicos. Recebeu alta hospitalar após 28 dias de internação assintomática do ponto de vista cardiovascular.
