# Supplementary material for: Paciente em Parada Cardiorrespiratória – É Possível a Realização de Implante Percutâneo de Valva Aórtica (TAVI) nesse Cenário?
Source: Arq Bras Cardiol. 2021 Aug 9;117(2):404–6. [Article in Portuguese] doi: 10.36660/abc.20201097 (PMC8395808; doi:10.36660/abc.20201097)
Supplement: Translation

# Translation: Patient in Cardiorespiratory Arrest - Is it Possible to Perform Transcatheter Aortic Valve Implantation (TAVI) in this Scenario?

**Authors:** Alexandre de Matos Soeiro, Francisco Akira Cardozo, Patrícia Oliveira Guimarães, Marcel Paula Pereira, Paulo Vinícius Ramos Souza, Gustavo A. B. Boros, Viviane Cordeiro Veiga, Samolon Soriano Ordinola Rojas, Fernanda Marinho Mangione, Salvador André Bavaresco Cristóvão, Gustavo Alexandre Dutra, Adnan Ali Salman, Luiz Eduardo Loureiro Bettarello, José Armando Mangione

**Affiliations:** 1 São PauloSP Brazil Hospital Beneficência Portuguesa de São Paulo - Unidade Cardiológica Intensiva, São Paulo, SP – Brazil

**Keywords:** Aortic Valve, Heart Arrest, Emergencies, Cardiogenic Shock

## Introduction

Transcatheter aortic valve implantation (TAVI) is a well-established procedure in reference centers worldwide and is currently accepted as the method of choice in high- and intermediate-risk patients. Although unusual, catastrophic complications can occur before or after its implantation, such as coronary obstruction, rupture of the aortic valve annulus, cardiac tamponade, significant perivalvular insufficiency and prosthesis embolization/displacement.

Data from a recently published North American registry showed that 1,695 patients (2.8%) undergoing TAVI required some type of mechanical circulatory support during hospitalization. It was observed that heart failure, transapical access, respiratory diseases, acute myocardial infarction, cardiorespiratory arrest (CRA) and cardiogenic shock were the factors most frequently associated with the need for mechanical circulatory support.^1^

Although its use is widespread, mainly in high-risk patients, and the main complications of the method and the underlying disease are already known, its use in CRA is not yet indicated and to date, rarely described in the literature.^1^ Implantation during CRA makes the technique difficult due to chest compressions, impossibility of assessing the immediate result and limited time for effective expansion of the prosthesis.

In 2013, the use of mechanical compression (AutoPulse) was described for the first time during the occurrence of CRA after the start of TAVI as a safe and effective alternative, allowing the procedure to be carried out until its completion.^2^ In 2014, another case report showed the possibility of deformation of the prosthesis after manual CPR, which progressed with significant paravalvular leak and subsequent death.^3^

Based on these possibilities of complications, there are protocols for indicating extracorporeal membrane oxygenation (ECMO) in patients undergoing TAVI who progress to cardiogenic shock or even CRA. In high-risk cases, some services have organized and managed to place patients who progress to CRA during TAVI on ECMO in up to 5 minutes. This allows the rapid stabilization of the patient before the occurrence of multiple-organ dysfunction and even carrying out the procedure to the end.^4^

Thus, reports of complications and CRA associated with TAVI have only been described during or after the endoprosthesis expansion procedure. The aim of the present case is to share the success and pioneering spirit of a Brazilian reference center in TAVI performance in a patient with pre-procedural CRA as a rescue measure.

## Description

An 84-year-old female patient had started having dyspnea at rest 6 days before. She reported a personal history of systemic arterial hypertension, dyslipidemia and acute myocardial infarction, at which point a percutaneous coronary intervention was performed with drug-eluting stent in the left main coronary artery and circumflex artery two months before. She was initially admitted via the emergency department of a secondary hospital. At admission, she was in poor overall health status, with tachycardia (heart rate = 120 bpm), tachypnea (respiratory rate = 28 bpm), blood pressure of 76x40 mmHg, and increased capillary refill time (6 seconds). Cardiac auscultation showed rhythmic heart sounds with systolic ejection heart murmur in aortic focus, +3/+6, and vesicular murmurs present with rales up to the middle third bilaterally on pulmonary auscultation. Initially, intravenous furosemide, norepinephrine and dobutamine were started in continuous infusion, in addition to the use of non-invasive ventilation.

The electrocardiogram did not show acute ischemic alterations and myocardial necrosis markers were negative. Additionally, an initial screening did not show any changes suggestive of an infectious condition. At that time, a transthoracic echocardiogram was requested, which showed a left ventricular ejection fraction of 28% with diffuse hypokinesia, calcified aortic valve, with a valve area of 0.6 cm^2^ and a maximum left ventricle-aorta gradient of 66 mmHg and a mean of 35 mmHg, in addition to moderate mitral regurgitation (Figure 1).

**Figure 1:** Transthoracic echocardiogram in pre-TAVI apical window showing a calcified aortic valve, with valvular area of 0.6 cm^2^ and maximum left ventricle-aorta gradient of 66 mmHg and mean gradient of 35 mmHg.

After two failed attempts at weaning the patient from vasoactive drugs, it was decided to transfer the patient to a tertiary center using intensive care air transport. After the initial assessment and based on the risk associated with the patient, transfemoral TAVI was indicated. After 72 hours in the high-complexity hospital, the patient showed worsening of cardiogenic shock and the need for a progressive increase in vasoactive drugs (dobutamine 20 *μ*g/kg/min and norepinephrine 0.3 *μ*g/kg/min). Therefore, the patient was urgently transferred to the hemodynamics laboratory to undergo the percutaneous procedure. The patient was placed in the horizontal decubitus position, and the orotracheal intubation, asepsis procedure and femoral access were performed. At the start of the transvenous pacemaker lead introduction, the patient showed CRA with ventricular fibrillation rhythm with rapid degeneration to asystole. Cardiopulmonary resuscitation (CPR) measures were initiated in accordance with advanced cardiology life support (ACLS) guidelines. At that moment, as a rescue measure, during CPR and without the aid of the echocardiogram, TAVI was performed with the Edwards SAPIEN 3 prosthesis N. 26 only with scope visualization in the room, with a total procedure time of 4 minutes (Figure 2). Immediately after that, the patient returned to spontaneous circulation rhythm. During the early post-procedure period, the patient progressed with worsening of cardiogenic shock, requiring increased doses of norepinephrine, use of vasopressin and intra-aortic balloon pump. A new transthoracic echocardiogram showed a left ventricular ejection fraction of 33% with diffuse hypokinesia, prosthesis in the aortic position, with a maximum left ventricle-aorta gradient of 35 mmHg and a mean gradient of 17 mmHg, with mild to moderate peri-prosthesis aortic insufficiency (Figure 3).

**Figure 2:** TAVI was performed with an Edwards SAPIEN 3 prosthesis n. 26, with scope visualization only, during CPR maneuvers. A) Positioning of catheters; B) Alignment of the prosthesis in the aortic valve annulus; C) Prosthesis shown in the aortic position.

**Figure 3:** Transthoracic echocardiogram after TAVI, showing a left ventricular ejection fraction of 33%, prosthesis in the aortic position with a maximum left ventricle-aortic gradient of 35 mmHg and a mean gradient of 17 mmHg, with mild to moderate peri-prosthesis aortic insufficiency.

After 48 hours, the patient showed improvement in hemodynamic parameters, and was progressively weaned from mechanical support and vasoactive drugs and submitted to extubation on the seventh day after TAVI, remaining without neurological deficits. She was discharged from the hospital after 28 days of an asymptomatic hospitalization from a cardiovascular viewpoint.
